# Pandemic Swine-Origin H1N1 Influenza Virus Replicates to Higher Levels and Induces More Fever and Acute Inflammatory Cytokines in Cynomolgus versus Rhesus Monkeys and Can Replicate in Common Marmosets

**DOI:** 10.1371/journal.pone.0126132

**Published:** 2015-05-06

**Authors:** Petra Mooij, Gerrit Koopman, Daniëlla Mortier, Melanie van Heteren, Herman Oostermeijer, Zahra Fagrouch, Rudy de Laat, Gary Kobinger, Yan Li, Edmond J. Remarque, Ivanela Kondova, Ernst J. Verschoor, Willy M. J. M. Bogers

**Affiliations:** 1 Department of Virology, Biomedical Primate Research Centre, Rijswijk, The Netherlands; 2 National Microbiology Laboratory, Public Health Agency of Canada, Winnipeg, Manitoba, Canada; 3 Department of Parasitology, Biomedical Primate Research Centre, Rijswijk, The Netherlands; 4 Animal Science Department, Biomedical Primate Research Centre, Rijswijk, The Netherlands; Johns Hopkins University - Bloomberg School of Public Health, UNITED STATES

## Abstract

The close immunological and physiological resemblance with humans makes non-human primates a valuable model for studying influenza virus pathogenesis and immunity and vaccine efficacy against infection. Although both cynomolgus and rhesus macaques are frequently used in influenza virus research, a direct comparison of susceptibility to infection and disease has not yet been performed. In the current study a head-to-head comparison was made between these species, by using a recently described swine-origin pandemic H1N1 strain, A/Mexico/InDRE4487/2009. In comparison to rhesus macaques, cynomolgus macaques developed significantly higher levels of virus replication in the upper airways and in the lungs, involving both peak level and duration of virus production, as well as higher increases in body temperature. In contrast, clinical symptoms, including respiratory distress, were more easily observed in rhesus macaques. Expression of sialyl-α-2,6-Gal saccharides, the main receptor for human influenza A viruses, was 50 to 73 times more abundant in trachea and bronchus of cynomolgus macaques relative to rhesus macaques. The study also shows that common marmosets, a New World non-human primate species, are susceptible to infection with pandemic H1N1. The study results favor the cynomolgus macaque as model for pandemic H1N1 influenza virus research because of the more uniform and high levels of virus replication, as well as temperature increases, which may be due to a more abundant expression of the main human influenza virus receptor in the trachea and bronchi.

## Introduction

Animal models play an important role in studying the pathogenesis of influenza virus infections [1,2]. While the majority of the studies are performed in mice and ferrets, other species including the rat, cat, dog, pig, guinea pig and non-human primates (NHP) have been shown to be susceptible to influenza A virus infection and were found to develop clinical signs and histo-pathological changes to varying degrees [1,2]. NHP are genetically closely-related to humans and show immunological and physiological resemblances to humans that make them a highly relevant model in pre-clinical safety, immunogenicity and efficacy evaluation of vaccines and therapies [3]. Several macaque species, including cynomolgus macaques (*Macaca fascicularis)*, rhesus macaques (*M*. *mulatta)* and pigtailed macaques (*M*. *nemestrina*) have been infected with human influenza A viruses [1,4–14]. Usually these viruses replicate in the upper respiratory tract and infections are either asymptomatic or cause mild clinical symptoms. However, acute respiratory distress and fatal outcome have been observed in these animal models after infection with avian H5N1 [4,13] and the 1918 H1N1 virus [15].

More recently, swine-origin H1N1 influenza A viruses from the early stage of the 2009 pandemic were shown to induce upper and lower respiratory tract infections and clinical disease in cynomolgus macaques [16,17], while no clinical symptoms were noted in rhesus macaques [18]. In addition, the common marmoset (*Callithrix jacchus*), a New World NHP species, was found to be susceptible to infection with the 2009 pandemic A/California/07/2009 virus and animal-to-animal transmission was noted [19]. Despite the many studies on influenza A virus infection in cynomolgus and rhesus macaques, a direct comparison between these species with regard to susceptibility to infection and clinical manifestations has not been made. Hence, it is not known which of these two species is the optimal animal model. Here we have used a recently characterized swine-origin influenza A/Mexico/InDRE4487/2009 virus strain, that was shown to result in upper and lower respiratory tract infection and clinical disease in cynomolgus macaques [17], to perform a head-to-head comparative study between cynomolgus versus rhesus macaques and common marmosets. We observed that all three monkey species were susceptible to infection and developed upper and lower airway infections. However, cynomolgus macaques showed higher levels and longer duration of virus replication in the tracheal swabs and lungs and developed higher fever, while clinical symptoms were more pronounced in rhesus monkeys. We observed 50–73 times higher expression levels of sialyl-α-2,6-Gal saccharides, the receptor for human influenza A virus, in the trachea and bronchi of cynomolgus macaques compared to rhesus macaques, which may have caused the differences in virus replication.

## Materials and Methods

### Animals

This study was performed in outbred mature Indonesian and Malaysian origin cynomolgus monkeys (*Macaca fascicularis*), Indian-origin rhesus monkeys (*M*. *mulatta*), and common marmosets (*Callithrix jacchus*). Animals were captive-bred for research purposes and socially housed at ABSL-III facilities at the Biomedical Primate Research Center, Rijswijk, The Netherlands (an AAALAC-accredited institution). Animal housing was according to international guidelines for non-human primate care and use (The European Council Directive 86/609/EEC, and Convention ETS 123, including the revised Appendix A as well the ‘Standard for humane care and use of Laboratory Animals by Foreign institutions’ identification number A5539-01, provided by the Department of Health and Human Services of the United States of America’s National Institutes of Health (NIH)). All animal handlings are performed within the Department of Animal Science (ASD) according to Dutch law. A large, experienced staff is available, including full-time veterinarians and a pathologist. ASD is regularly inspected by the responsible authority (Voedsel en Waren Autoriteit, VWA), and by an independent Animal Welfare Officer. The animals were negative for antibodies to SIV, simian type D retrovirus, simian T-cell lymphotropic virus, and were selected for absence of antibodies directed to conserved nucleo- and matrix proteins covering all human and avian Influenza A and B viruses (Serion ELISA classic Influenza A/B virus IgA/IgG/IgM detection kit (ESR 1231, Serion immunodiagnostica GmbH, Würzburg, Germany)) and to influenza A/PR/8/34 (H1N1) viral lysate (Advanced Biotechnologies Inc, Eldersburg MD, USA). All animals were classified healthy according to physical examination and evaluation of complete blood count and serum chemistry.

During the experiment the animals were pair-housed with a socially compatible cage mate. Animals were kept on a 12-hour light/dark cycle and were housed in a single room separated from the rest of the colony. The monkeys were offered a daily diet consisting of monkey food pellets (Hope Farms, Woerden, The Netherlands), fruit and bread. Enrichment was provided daily in the form of pieces of wood, mirrors, food puzzles, a variety of other homemade or commercially available enrichment products. Drinking water was available *ad libitum* via an automatic watering system. Veterinary staff provided daily health checks before infection, and the animals were checked for appetite, general behavior and stool consistency. During the course of the influenza virus infection the animals were checked twice a day, and scored for clinical symptoms according to a previously published scoring system [20], such as skin and fur abnormalities, posture, eye and nasal discharge, sneezing and coughing, respiration rate. A numeric score of 35 or more was predetermined to serve as an endpoint and justification for euthanasia. Each time an animal was sedated the body weight was measured. Body temperature was recorded on a data storage tag (DST, Micro-T, Star-Oddi, Iceland) surgically placed in the abdominal cavity of each animal 28 days before infection, recording body temperature every 15 minutes. All steps were taken to insure the welfare and to avoid any suffering of the animals. All experimental interventions (intra-bronchial infection, swabs, blood samplings) were performed under anesthesia using ketamine. Before euthanasia, animals were first sedated deeply with ketamine, and subsequently euthanized by intra-cardiac injection of an overdose of pentobarbital.

The Institutional Animal Care and Use Committee of the Biomedical Primate Research Centre (dierexperimentencommissie, DEC-BPRC), approved the study protocols developed according to strict international ethical and scientific standards and guidelines (DEC advice #689). The qualification of the members of this committee, including their independence from a research institute, is requested in the Dutch law on animal Experiments (Wet op de Dierproeven, 1996).

### Experimental infection

Six male adult animals from each species were experimentally inoculated with 0.5x10^6^ (marmoset) or 4x10^6^ (cynomolgus and rhesus) TCID_50_ of an influenza A/Mexico/InDRE4487/2009 (H1N1) (Mex4487) virus stock that was produced on Madin-Darby Canine Kidney (MDCK) cells. This virus had been isolated from the bronchial aspirate of a 26-year-old man from a family cluster of three confirmed severe flu cases in Mexico [17]. Cynomolgus and rhesus macaques were infected with 2 ml of an influenza virus suspension containing 10^6^ TCID_50_/ml in each lung lobe via the intra-bronchial route using a bronchoscope. Common marmosets were infected through the intra-tracheal route using a catheter inserted into the trachea followed by injection of 0.5 ml influenza virus at a concentration of 10^6^ TCID_50_/ml.

During the infection procedure, animals were sedated with ketamin (10 mg/kg). Additionally, they received medetomidine hydrochoride, 0.04 mg/kg (Cepetor) to induce further sedation and muscle relaxation. At the end of the procedure Atipamezol hydrochloride, 0.5 mg/kg (Revertor) was used for faster recovery. Local anesthesia in the throat was applied by spraying with 10% Xylocain (Lidocain).

From each species 2 animals (cage mates) were sacrificed on day 3, two on day 6 and two on day 14. Before euthanasia bronchoalveolar lavages (BAL) were collected from cynomolgus and rhesus macaques. Tissues collected at necropsy (conjunctiva, oro/nasopharynx, nasal mucosa, trachea, tonsil, right and left bronchus, 6 lung lobes, bronchial lymph node, heart and jejunum) were processed to assess virus distribution in the tissues and for lung histopathology. Blood, tracheal and nasal swabs (macaques), or throat swabs (marmoset), were collected on days 0, 1, 2, 3, 4, 6, 8, 10, and 14. Swabs were taken using Copan flocked swabs (FLOQswabs, 502CS01).

### Radiographic evaluation

Thoracic radiographs were taken from 2 sides of the lungs (ventro-dorsal and left lateral) on days 0, 1, 2, 3, 4, 6, 8, 10, and 14 after infection using a portable x-ray apparatus (HF 80 ML, Gierth GmbH, Germany). The digital images were interpreted by an independent radiologist with OsiriX software and were scored as follows: grade 0, normal examination; grade 0.5, suspected interstitial pulmonary infiltrates, grade 1, mild interstitial pulmonary infiltrates; grade 2, moderate interstitial pulmonary infiltrates, possibly including partial cardiac border effacement and small areas of pulmonary consolidation; and grade 3, pulmonary consolidation as primary lung pathology, often seen as progression from grade 2 lung pathology, according to Brining et al. [20].

### Lung pathology

Lung tissues taken at necropsy were fixed in 10% formalin, processed by conventional methods and embedded in paraffin. 4μm sections were prepared and stained with hematoxylin and eosin according to standard procedures.

### Influenza virus replication

Virus replication was monitored by standard TCID_50_ assays on MDCK cells. The detection limit of this assay was 100 infectious viral particles/ml. Viral RNA was detected by real-time PCR, as described by Poon *et al*. [21]. Viral RNA was isolated using a QIAamp Viral RNA Mini kit (Qiagen Benelux BV, Venlo, The Netherlands) following the manufacturer's instructions. The assay was carried out using the Brilliant QRT-PCR Core Reagent Kit, 1-Step (Stratagene Europe, Amsterdam, The Netherlands) in a 25 μl volume with final concentrations of 160 nM for each primer, 200 nM for the probe, 5.5 nM MgCl2, and using 10 μl RNA, extracted from 140 μl sample volume. RNA was reverse transcribed for 30 min at 50°C. Then, after a 10 min. incubation step at 95°C, the cDNA was amplified for 40 cycles, consisting of 30 sec. denaturation at 95°C, followed by a 1 min. annealing-extension step at 60°C. All the reactions were carried out with an iQ5 Multicolor Real-Time PCR Detection System (Bio-Rad Laboratories BV, Veenendaal, The Netherlands). The detection limit of the assay was 20 RNA copies per reaction. Each sample was analyzed 2–3 times.

Virus concentrations were determined in blood, swabs, BAL and tissues, collected as described under “experimental infection”. Tissues were homogenized in 2 ml MEM in M-tubes of a GentleMACS dissociator (Miltenyi Biotec GmbH, Bergisch Gladbach, Germany) using protein-1 protocol. Homogenized tissue was centrifuged and supernatant was put through a cell strainer of 40 μm. Supernatant was tested for the presence of viral RNA as described. Swabs were placed in 2 ml MEM, supplemented with 0.5% bovine serum albumin (BSA), fungizon (2.5 μg/ml), penicillin (100 U/ml) and streptomycin (100 μg/ml). After vortexing the swab was removed and the samples were stored at -20°C.

### Hematology and FACS analysis

Hematology parameters were measured on EDTA blood with a Sysmex XT-2000iV Automated Hematology Analyzer (Sysmex Corporation of America). Clinical chemistry parameters were measured in serum samples with a Cobas Integra 400 plus machine (Roche Diagnostics, Burgess Hill, United Kingdom). FACS analysis was performed on blood as described previously [22]. The following monoclonal antibody (mAb) combinations were used: a) CD20^V450^, CD3^V500^, CD8^BV570^, CD45^FITC^, CD16^PE^, CD14^PE-TxRed^, HLA-DR^PerCP^, CD4^PE-CY7^, CD69^APC-CY7^, b) CD3^V500^, CD8^BV570^, Ki67^FITC^, CD25^PE^, CD28^PE-TxRed^, CD45RA^biot^, CD4^PE-CY7^, CD152^APC^. All mAbs were from Becton Dickinson, except for CD14 (Beckman Coulter, Brea, CA, USA) and CD8 (Biolegend, San Diego, CA, USA). Flow cytometry was performed on a FACSaria machine using Diva software (Becton Dickinson, Franklin Lakes, NJ, USA). For each tube ≥30,000 events in the lymphocyte-gate were recorded.

### Assessment of cytokine and chemokine protein levels in serum

Cytokine and chemokine concentrations, including G-CSF, GM-SCF, IFNγ, IL-1β, IL-1Ra, IL-2, IL-4, IL-6, IL-8, IL-12, IL-13, IL-15, IL-17, IL-18, MCP-1, MIP-1α, MIP-1β, sCD40L, TGFα, TNFα, were determined using the MILLIPLEX MAP Non-Human Primate Cytokine Magnetic Bead Panel kit (Millipore, Billerica, MA, USA) and a Luminex detection system (Luminex Corperation, Austin, USA), according to the manufacturer’s instructions. Detection of these cytokines was validated by the manufacturer, showing equal cross reactivity for cynomolgus and rhesus macaques. For each cytokine a standard curve was made, ranging from 2.4 to 10,000 pg/ml. Samples were measured on a Bio-Plex 2000 system (Bio-Rad, Herts, UK) and analyzed by using Bio-Plex Manager software.

### Quantification of sialyl-α-2,6-Gal saccharides in tissue samples

Trachea, bronchus and lung tissues were cut into small pieces, and 0.5 to 1 ml PBS containing 0.5 mM phenyl-methyl-sulfonyl fluoride (PMSF) as protease inhibitor, was added. Tissues were disrupted in a Mini-Bead-Beater-24 (Biospec Products, Bartlesville, OK, USA), using a 2 ml microvial with three 3.2 mm chrome steel beads, by performing 5 times pulsing for 3 minutes at full power. The homogenate was then centrifuged 30 minutes at 5000 x g at room temperature. Sialidase treatment as a negative control for specific detection of sialyl-α-2,6-Gal saccharides was performed by incubating 250 μl homogenate with 200 U neuraminidase (New England Biolabs, Ipswich, MA, USA) for 16 hours at 37°C under rotation (30 rpm). Subsequently, samples were treated with 1% sodium dodecyl sulphate (SDS) for 16 hours at 4°C and then heated at 95°C for 10 minutes. Gel electrophoresis was performed on a 4–12% Nu-PAGE gel (Life technologies, Carlsbad, CA, USA) using Nu-PAGE MES SDS running buffer (Invitrogen, Carlsbad, CA, USA) for 1 hour at 150 Volt. Samples were blotted on pre-wetted polyvinyl difluoride (PVDF) membranes (Bio-Rad) and after 1 hour blocking at room temperature with carbon-free blocking buffer (Vector Labs, Burlingame, CA, USA) the blots were incubated with biotinylated *Sambucus nigra* agglutinin (SNA) (Vector Labs) at 1:100 dilution in 10% carbon-free blocking buffer, overnight at 4°C. After washing with PBS-Tween20 (0.05%) the blots were incubated with a streptavidin-biotin-peroxidase complex (Vectastain ABC-kit, Vector Labs), and the color reaction was developed with diamino-benzidine (Sigma-Aldrich, MO, USA). SeeBlue Plus2 pre-stained standard (Invitrogen) was used as molecular range marker, and a concentration range of Fetuin from bovine serum (Sigma-Aldrich) was used as standard for sialyl-α-2,6-Gal saccharides content. Staining intensity was measured on a ChemiDoc MP imaging system (Bio-Rad) and analyzed using Image Lab software (Bio-Rad). Staining intensity in the tissue samples is expressed as signal of the 56 kD band per gram tissue as percentage of the signal of the 62 kD band in Fetuin (per gram Fetuin). These bands were selected for analysis, because they were clearly separated from the other bands and thus easier to quantify. In addition, the decrease in staining intensity upon dilution of the sample was similar for all bands in the tissue samples and for all bands in Fetuin.

### Statistical evaluation

Statistical significance of any difference between species was calculated by using the Mann-Whitney rank sum test. A two-sided α level of 0.05 was used to determine significance.

## Results

### Rhesus macaques develop more distinct clinical symptoms than cynomolgus macaques and common marmosets during infection with influenza virus Mex4487

**Fig 1:**
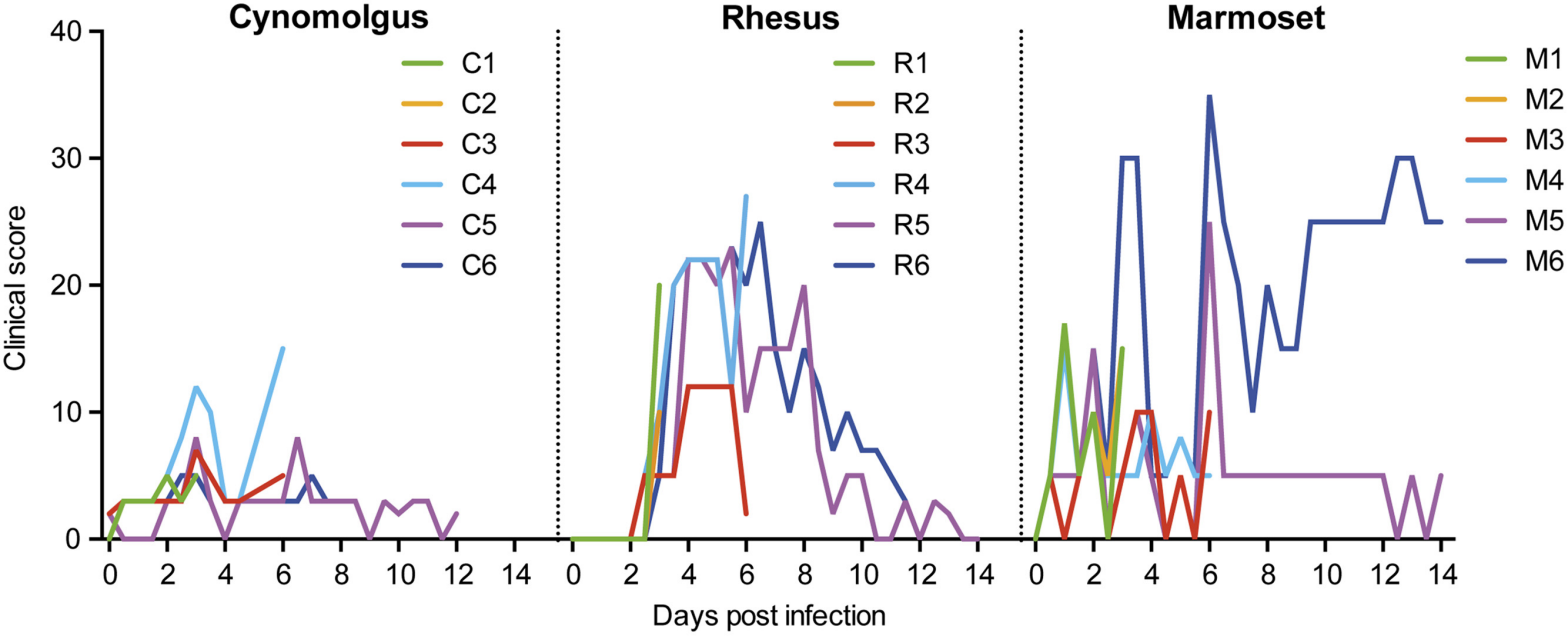
Clinical score of cynomolgus macaques, rhesus macaques, and common marmosets. For each individual animal the total clinical score is shown in time after infection with Mex4487 influenza virus at day 0.

Using the clinical scoring system previously described by Brining et al [20], rhesus monkeys developed more clinical symptoms during the first 6 days after infection compared to cynomolgus macaques (Fig 1). Rhesus monkeys showed reduced appetite, an unkempt appearance, hunched posture and labored breathing, starting at day 3 post-infection. By day 9 the animals had largely recovered. In contrast, cynomolgus macaques showed a slight loss of appetite during the first week after infection. In common marmosets the pattern was more variable (Fig 1). Three out of six animals displayed labored breathing at day 1 after infection and all animals showed a total loss of appetite at day 2. Most of the animals recovered over the next few days, except for animal M5, who displayed sudden fast breathing and inactivity at day 6 after infection, and animal M6 who had persistent fast breathing and displayed tremors. Sneezing, nasal discharge or coughing was almost never observed in either cynomolgus or rhesus macaques or common marmosets. There was no weight loss associated with influenza virus infection (data not shown).

### Cynomolgus macaques develop higher virus loads and higher fever than rhesus macaques during Mex4487 infection

**Fig 2:**
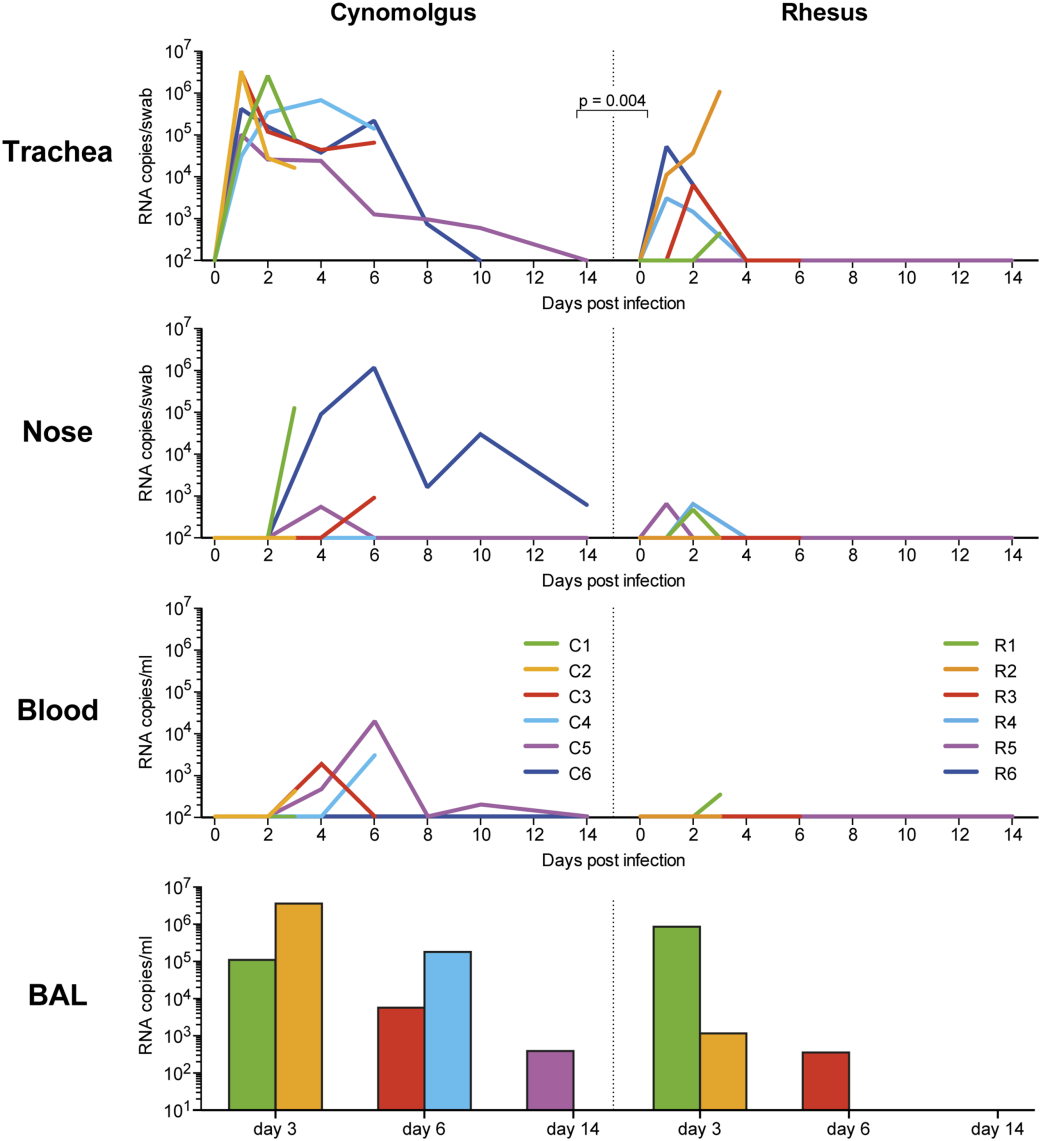
Virus replication in cynomolgus macaques and rhesus macaques after Mex4487 influenza virus infection. Virus load measured by RT-PCR in tracheal swabs, nose swabs and serum in time and in bronchoalveolar lavage (BAL) fluid taken just before necropsy; i.e. at day 3, 6 and 14 in two animals each time, measured in cynomolgus and rhesus monkeys. Colors in the BAL bar graphs correspond to colors in the line graphs. P = 0.004: statistically significant difference in virus load in tracheal swab. Viral load was calculated for each animal as area under the curve (AUC). For statistical analysis the AUC in six cynomolgus macaques were compared with six rhesus macaques (Mann-Whitney test).

After inoculation, replicating influenza virus was detected in all 3 non-human primate species. Virus levels, as detected by PCR, were highest in the tracheal swabs followed by nasal swabs, and occasionally viral RNA was detected in blood samples (Fig 2). In the tracheal swabs, virus was detected in all 6 cynomolgus and in 5/6 rhesus macaques. Virus production was significantly higher (P = 0.004) and detectable for a longer time period in cynomolgus macaques than in rhesus macaques. In marmosets, tracheal swabs could physically not be taken. In this species throat swabs were taken, possibly explaining why, in contrast to the tracheal swabs, only occasionally a positive signal could be detected (in marmoset M3 1.2x10^4^ RNA copies/ml on day 6, in M4 3.4x10^4^ RNA copies/ml on day 6, and in animal M6 8.9x10^7^ RNA copies/ml on day 8; S1 Table).

**Fig 3:**
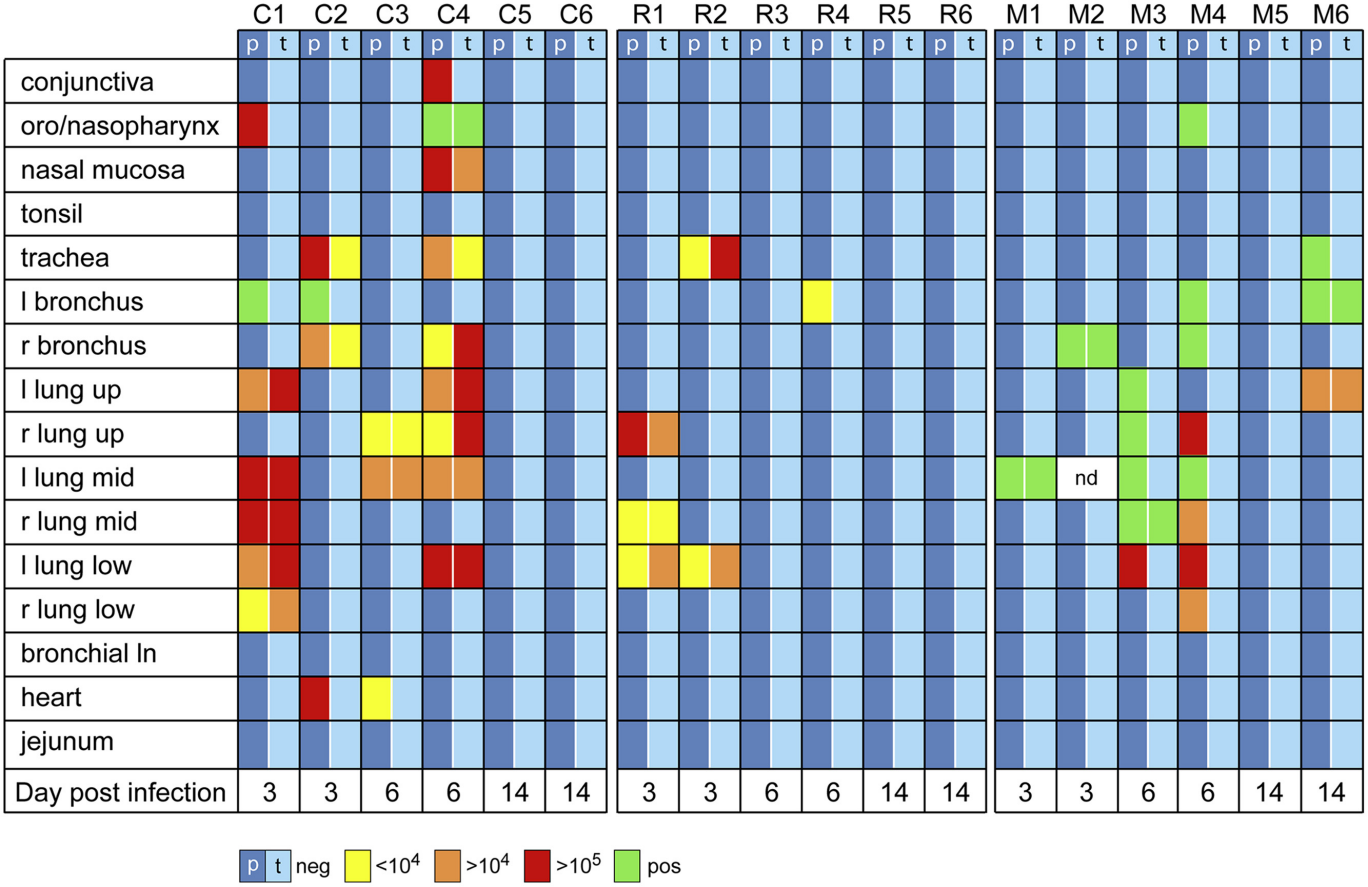
Virus detection in tissues of cynomolgus macaques, rhesus macaques and common marmosets after Mex4487 influenza virus infection. Virus detection in tissues taken at time of autopsy, performed on two animals from each species at day 3, 6, and 14 after infection. Shown are the virus loads (as RNA copies/gr tissue and as TCID_50_/gr tissue) indicated in different colors (blue: negative, yellow: <10^4^, orange: 10^4^—10^5^, red: >10^5^ RNA copies/gr tissue). Indicated in green are positive samples that could not be reliably quantitated, because they were below the cut-off of our assay (20 vRNA copies per reaction). nd: not done, C: cynomolgus, R: rhesus, M: marmoset, l; left, r; right, ln; lymph node, p: RT-PCR, t: TCID_50_ on MDCK cells.

Similar differences between cynomolgus and rhesus macaques were observed in the lung lavages (Fig 2). Lung lavages were technically not possible in marmosets. In a range of tissues taken at necropsy, including six lung lobes, trachea and bronchus, influenza virus could be detected at day 3 and 6 after infection in all species and in one marmoset (M6) even at day 14 post-infection (Fig 3). This animal M6, also showed numerous clinical symptoms until the end of the study, as shown in Fig 1. Rhesus macaques showed fewer virus-positive tissues than cynomolgus macaques at day 3 and 6 after infection (Fig 3). Replicating capacity of influenza virus found in PCR-positive samples (trachea/throat swabs, nose swabs, blood, BAL and necropsy tissues) was confirmed by tissue culture on MDCK cells (Fig 3 and S1 Table).

Body temperature was recorded every 15 minutes on a data logger (Star-Oddi) surgically implanted in the abdominal cavity of each animal (28 days before infection) and removed at necropsy. This method of body temperature registration allowed accurate measurements throughout the study period without sedation, obtaining valuable information that would have otherwise been lost.

**Fig 4:**
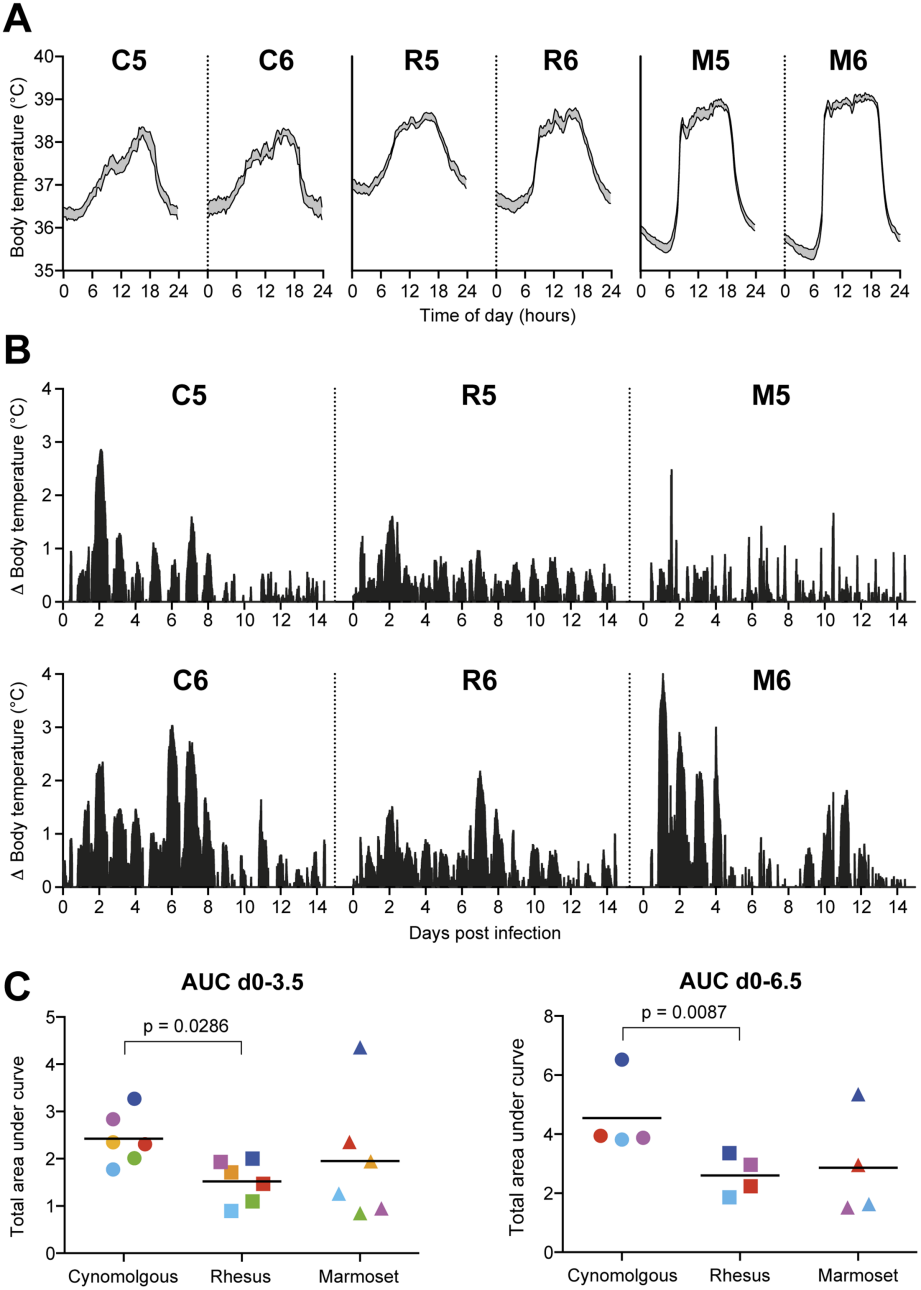
Body temperature of cynomolgus macaques, rhesus macaques and common marmosets before and after Mex4487 influenza virus infection. (A) Circadian temperature pattern, shown for two animals of each species (C5, C6, R5, R6, M5 and M6). The circadian pattern was calculated from the temperatures recorded during three weeks before infection. Grey areas represent the mean temperature with the 95% confidence interval. (B) Body temperature increase during infection. Shown is the net-increase in temperature, which was calculated by subtracting the individual circadian body temperature from the actually recorded temperature in time after infection. Data are depicted from the two animals of each species that were maintained in study for 14 days (C5, C6, R5, R6, M5 and M6). (C) Cumulative net temperature increase, calculated as area under the curve (AUC) from the net-increase data, either for the first 3.5 days or the first 6.5 days of infection. Colored dots represent individual animals (green: CRM1, orange: CRM2, red: CRM3, light blue: CRM4, purple: CRM5, dark blue: CRM6). C: cynomolgus, R: rhesus, M: marmoset. Statistical differences between groups were determined with Mann-Whitney test.

Each animal showed a clear circadian body temperature pattern, with lowest temperatures around midnight and highest around 15:00 in the afternoon (Fig 4A). The mean 24 hour circadian pattern was calculated by taking the mean of the body temperatures recorded at the same time of the day during a period of 3 weeks before infection. In Fig 4A the mean of the recorded pre-infection body temperatures with a 95% confidence interval is shown in grey. The difference between the day and night body temperature was larger in marmosets than in macaques (Fig 4A), most probably because of the smaller size of marmosets.

Each animal developed a fever, defined as increase in temperature above the circadian pattern recorded before infection, that peaked around day 2 after infection (Fig 4B). Some animals showed a second fever peak around day 7 (cynomolgus and rhesus) or around day 11 (marmoset). The increase in body temperature after infection was significantly higher in cynomolgus macaques than in rhesus macaques (AUC of net temperature, circadian temperature subtracted, Fig 4C) during the first 3.5 days (p = 0.0286) and 6.5 days after infection (p = 0.0087). At later time points after infection only two animals were left per species and no statistical evaluation was possible. Body temperatures of infected marmosets showed more individual variation and it was impossible to demonstrate statistically significant differences with the other species.

### Radiographic scoring and lung pathology

**Fig 5:**
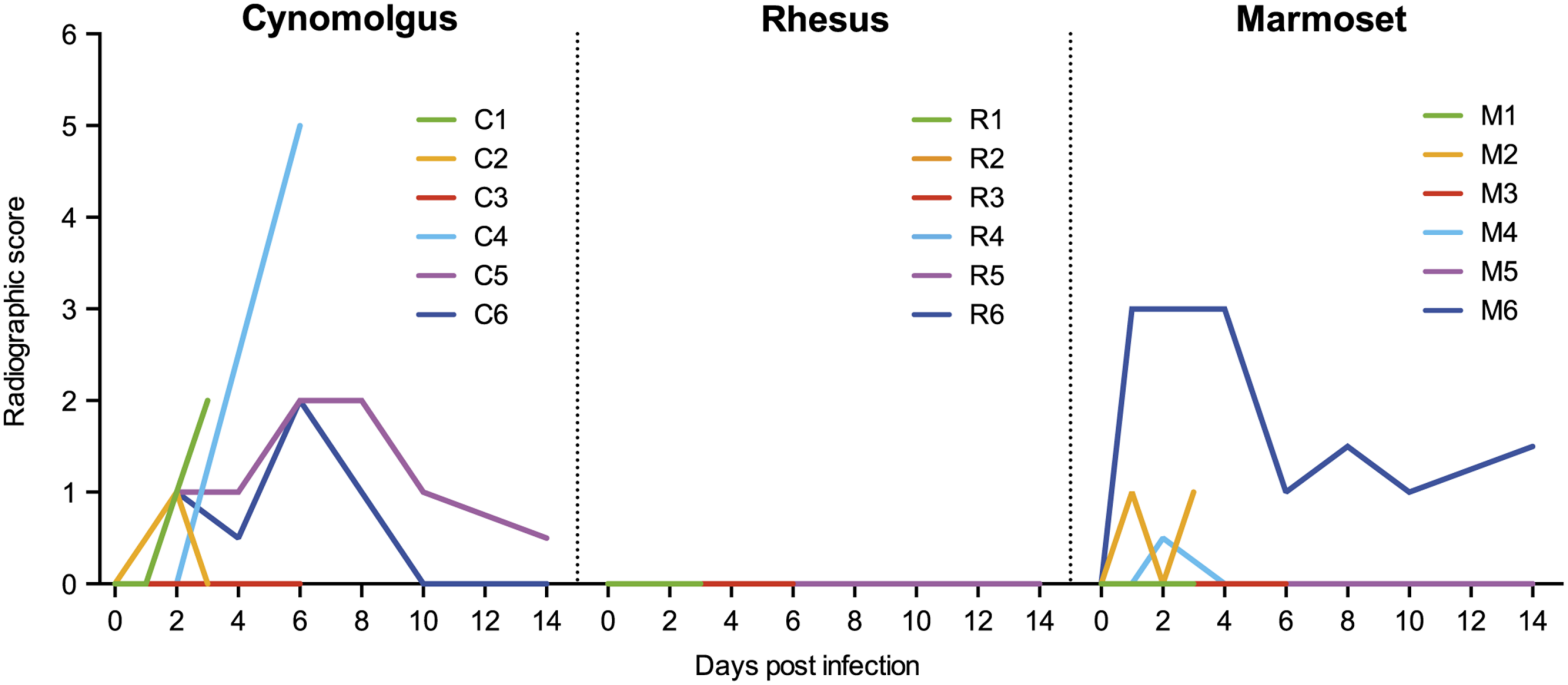
Radiographic score in cynomolgus macaques, rhesus monkeys and common marmosets in time. Both left and right lung lobes were scored from 0–3, giving a maximal radiographic score of 6 of the complete lung.

Respiratory disease development was further analyzed by radiographic imaging (X-ray) using a semi-quantitative scoring system. The first signs of pulmonary infiltration were observed on day 1 post-influenza virus infection in the lungs of two cynomolgus macaques (C2, C6) and two common marmosets (M2, M6). This developed further into mild (grade 1) to moderate (grade 2) interstitial pulmonary infiltrates with partial cardiac border effacement and small areas of pulmonary consolidation at day 6 of infection, while subsequently the severity of pulmonary infiltrates declined (Fig 5). Five out of six cynomolgus macaques (C1, C3, C4, C5, C6) and three out of six common marmosets (M2, M4, M6) showed different grades of pulmonary infiltrates at some point of influenza virus infection (S1 Fig, representative radiographs). Surprisingly, pulmonary infiltrates in rhesus macaque lungs could not be detected by X-ray examination, although they showed labored breathing as one of the clinical symptoms.

Two animals from each species were euthanized for necropsy on days 3, 6 and 14 post infection. Day 3 specimens were collected to study early disease progression and host responses, day 6 for maximum pathology and day 14 was chosen to examine recovery from infection.

Gross pathological examination revealed moderate multifocal pulmonary consolidation with hemorrhage, edema, and enlarged tracheobronchial lymph nodes. The lungs of the animals euthanized on 14 days post-infection (dpi) exhibited milder and focally extensive similar lesions. No lesions were seen in the extra-respiratory tissues. The pulmonary gross lesions of marmosets that were euthanized at day 3, 6 and 14 were variable, and included multifocal hemorrhage, edema and consolidation. Histologically, areas of pulmonary consolidation corresponded with various phases of alveolar damage. The alveolar damage was characterized by areas of excessive proteinaceous fluid in the alveolar lumina (intra-alveolar edema), and the presence of intra-alveolar hemorrhages, random hyaline membrane formation, multifocal epithelial loss (necrosis), and the accumulation of variable number of neutrophils, macrophages and lymphocytes in the alveolar lumina and walls (S2 Fig, representative histological images). These findings are in accordance with earlier findings in cynomolgus macaques [17]. The histological pulmonary lesions of the rhesus macaques were more exudative, comprised of marked edema, hemorrhage and congestion, in comparison to the cynomolgus macaques in which the inflammatory cellular infiltrates were more prominent. This finding is confirmed by the radiographic observations. Accompanied lesions were comprised of moderate bronchiolitis, tracheitis and in few animals oro-pharyngitis with mild degenerated epithelium in the nasal mucosa. In all species the histological pulmonary lesions were less prominent and milder at day 14 post-infection compared to day 3 and 6 post infection.

Analyses on lung tissues from the marmosets were limited because of an incomplete data set due to tissue damage induced by intra-cardiac injection during euthanasia.

### Cynomolgus macaques express higher levels of sialyl-α-2,6-Gal saccharides in trachea and bronchus

**Fig 6:**
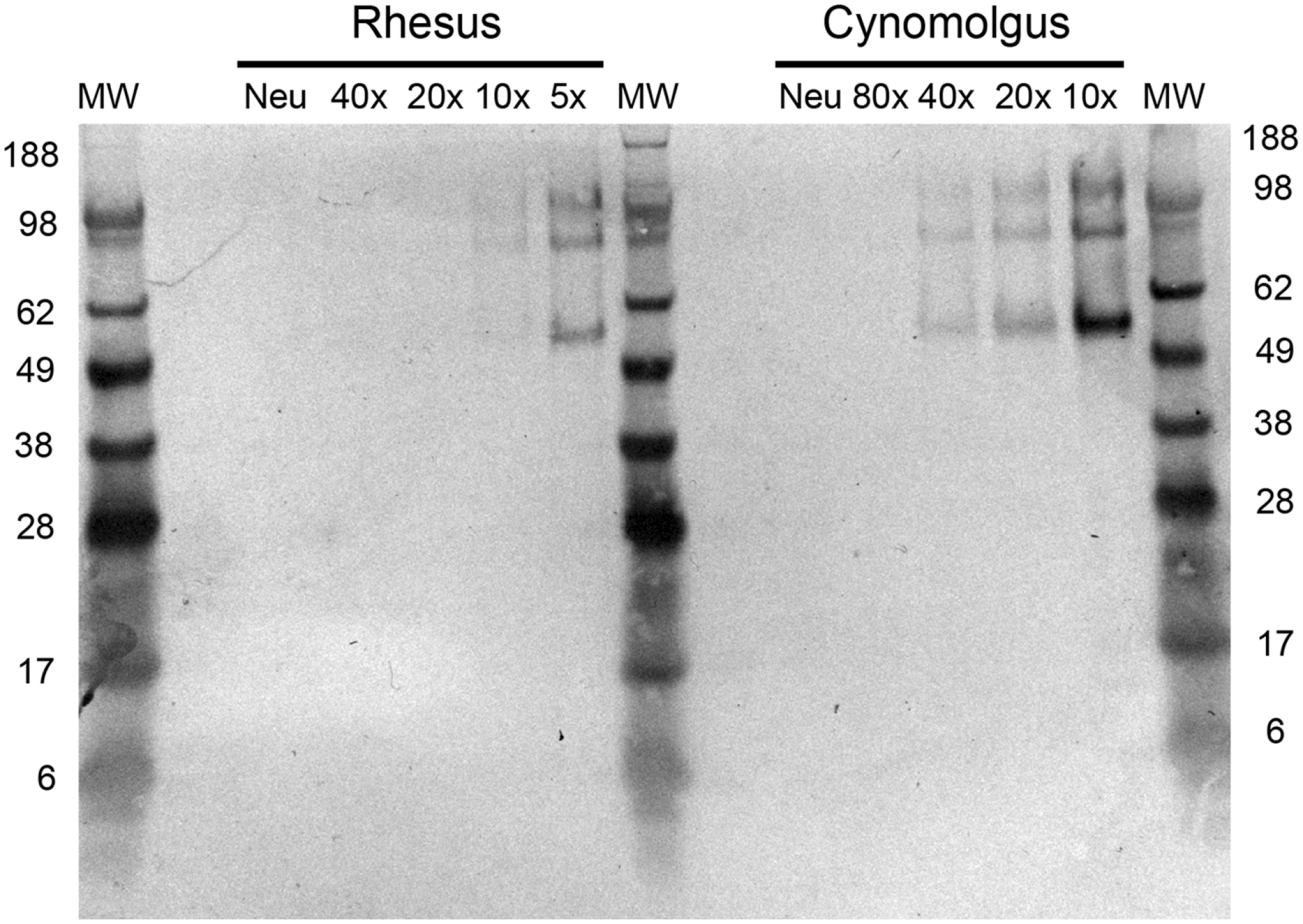
Levels of sialyl-α-2,6-Gal saccharides in trachea tissue samples from rhesus and cynomolgus monkeys. SNA stained blot of SDS-PAGE separated trachea tissue samples from rhesus and cynomolgus macaques applied in a dilution range of 1:5/1:10/1:20/1:40/1:80 or at a 1:5/1:10 dilution after neuraminidase digestion (Neu). The samples used were a mixture of tissue homogenates from 4 animals. MW = molecular weight marker. Numbers on the left and right side of the gel indicate the molecular weight (kD) of the marker bands.

Sialyl-α-2,6-Gal saccharides form the main receptors for human influenza A viruses. In order to evaluate if the observed differences in virus replication could be related to differences in expression of sialyl-α-2,6-Gal saccharides we analyzed tissue samples by SDS-PAGE followed by staining of bands with *S*. *nigra* agglutinin (SNA). As shown in Fig 6, a clear 56 kD band and several bands in the 80 to 120 kD MW range were visible in trachea tissue samples of cynomolgus and rhesus macaques upon SNA staining. Similar bands were observed in bronchus tissue (not shown). By using a dilution range the intensity of all bands was shown to be higher in cynomolgus than in rhesus monkeys. Measuring the density of the 56 kD band, which was present in all tissue samples and relatively clearly discernable, and expressing it as percentage of the signal of the 62 kD band in Fetuin, showed 50-fold higher expression in trachea (6.64 ± 2,42% versus 0.13 ± 0.02% of Fetuin) and 74-fold higher expression in bronchus tissue (5.57 ± 0.60% versus 0.08 ± 0.04% of Fetuin) of cynomolgus relative to rhesus monkeys (Table 1). Neuraminidase treatment was used as control for specific staining with SNA. Staining with *M*. *amurensis* agglutinin, which binds to sialyl-α-2,3-Gal and is used more preferentially by avian influenza viruses, only gave an indistinct broad smear and no clear separate bands could be discriminated. This made accurately quantification of this receptor not possible.

**Table 1:** Quantification of intensity of 56 kD band in SNA staining on SDS-PAGE of trachea and bronchus tissue samples from cynomolgus and rhesus monkeys.

	Trachea		Bronchus	
Animal	cynomolgus	rhesus	cynomolgus	rhesus
1	7.70	0.11	5.51	0.06
2	9.43	0.16	6.17	0.14
3	5.51	0.12	5.84	0.06
4	3.90	0.13	4.77	0.05
Mean ± SD	6.64 ±2.42	0.13 ± 0.02	5.57 ± 0.60	0.08 ±0.04

Indicated is the intensity of signal as percentage of signal from 62kD band of Fetuin (positive control).

### Decrease of neutrophilic granulocytes and activation of T-cells upon influenza virus Mex4487 infection in cynomolgus and rhesus macaques

**Fig 7:**
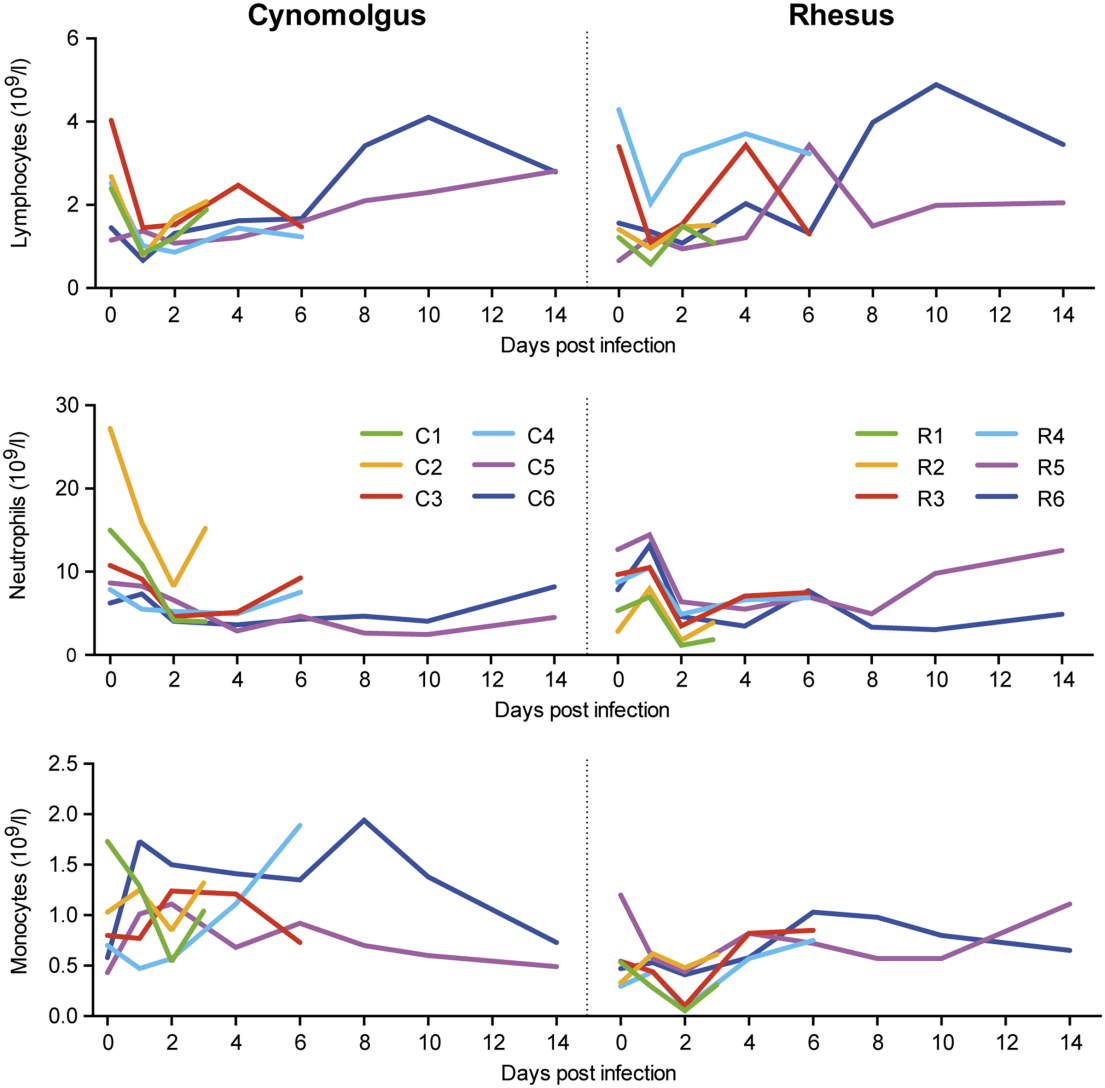
Lymphocyte, neutrophil and monocyte counts in blood of cynomolgus and rhesus macaques after Mex4487 influenza virus infection. Shown are the absolute counts (10^9^ cells/liter whole blood) for each individual animal in time after infection with Mex4487 influenza virus at day 0.

Evaluation of circulating white blood cells upon influenza virus infection revealed a prominent and early decrease in the number of lymphocytes and neutrophilic granulocytes, lowest counts being reached at day 1 and 2 respectively, both in cynomolgus and rhesus macaques (Fig 7). The effect on influenza virus infection on the number of circulating monocytes was more complex, with increases and decreases in animals from both species.

**Fig 8:**
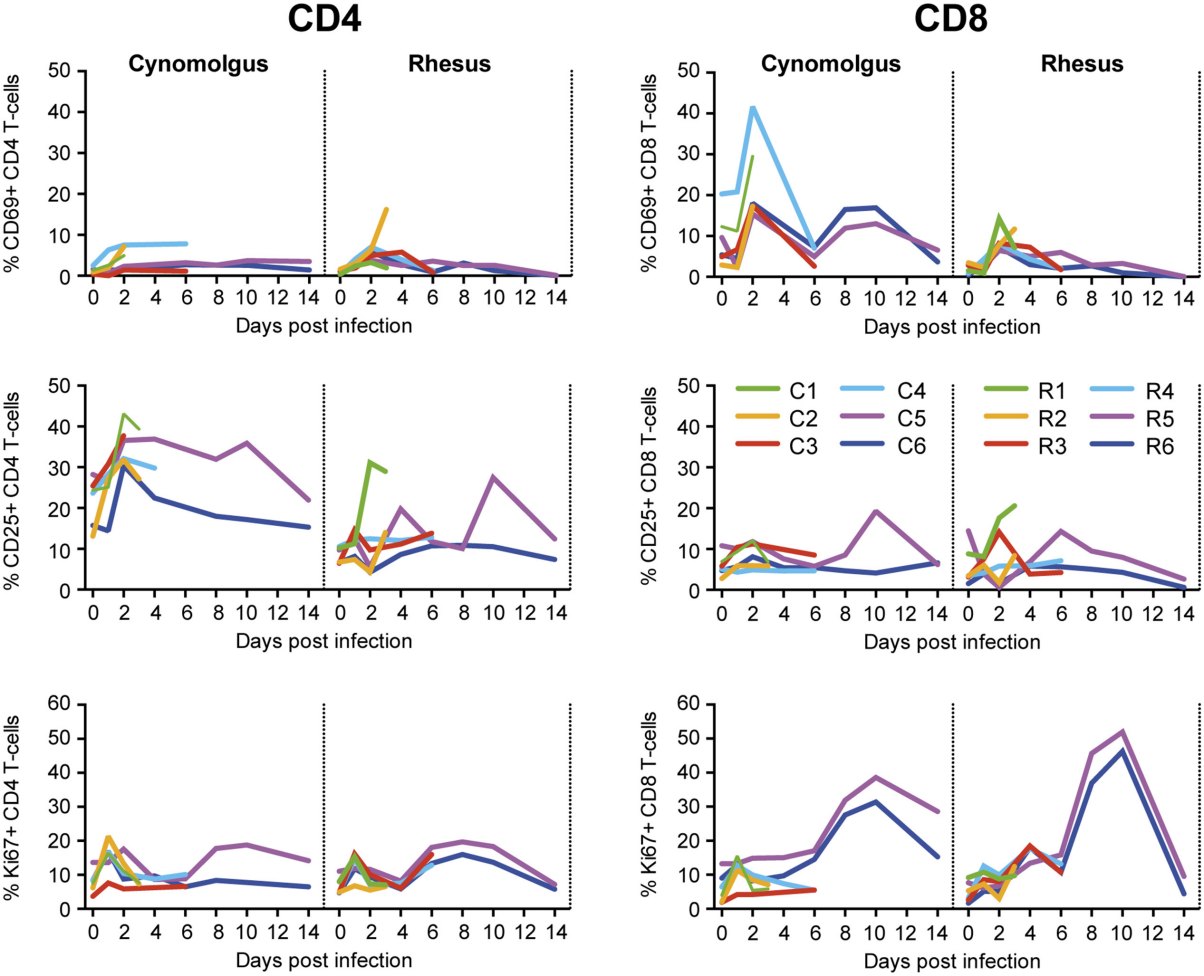
T-cell activation in cynomolgus and rhesus macaques after Mex4487 influenza virus infection. Shown is the percentage of CD4 T-cells (left graphs) and CD8 T-cells (right graphs) expressing CD69, CD25 or Ki-67 for each individual animal in time.

Detailed analysis of circulating CD4 and CD8 T-cells by FACS showed an early activation of both CD4 (measured by CD25 expression) and CD8 (measured by CD69 expression) T-cells, especially in cynomolgus macaques (Fig 8). T-cell proliferation, as determined by measuring Ki-67 expression, was observed at a low level in some animals immediately after infection, but was more prominent later after infection, particularly in CD8 T-cells, in both cynomolgus and rhesus macaques. Proliferation peaked at day 10 after infection and probably reflects the induction of an adaptive immune response. Similar measurements could not be performed in common marmosets because of the limited time points of blood collection.

### Cytokine and chemokine expression after influenza virus Mex4487 infection

**Fig 9:**
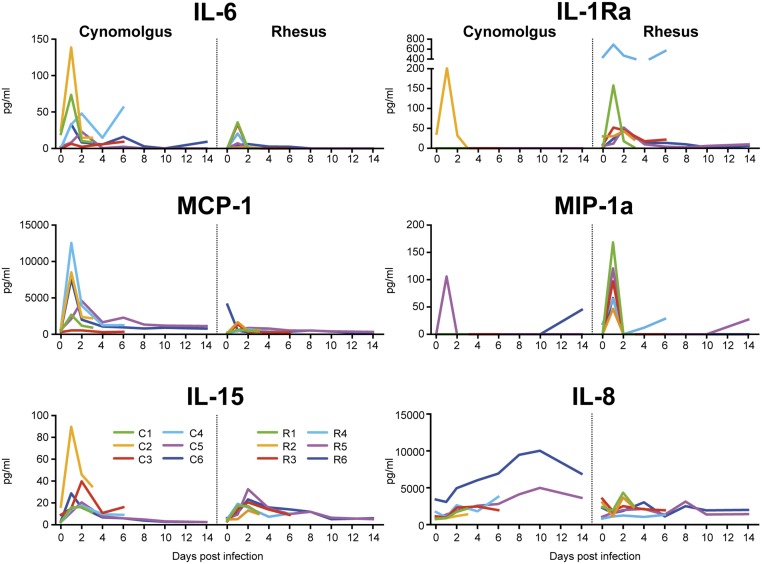
Cytokine and chemokine expression levels in serum of cynomolgus and rhesus macaques after Mex4487 influenza virus infection. Shown is the amount of IL-6, MCP-1, IL-15, IL-1Ra, MIP-1α or IL-8 in pg/ml in serum, for each individual animal in time. In each graph cynomolgus macaques are depicted left and rhesus macaques right.

Infection with Mex4487 influenza virus induced a strong increase in IL-6 and MCP-1 in the blood in cynomolgus macaques at day 1 after influenza virus infection and a gradual increase in IL-8 (Fig 9). In contrast, rhesus macaques rather showed an increase in IL-1Ra and MIP-1α, while IL-6 was only modestly increased, and MCP-1 remained low. A moderate increase in IL-15 was observed in both species 1–2 days after influenza virus infection (Fig 9). Other cytokines analysed were either below the detection limit of 2.4 pg/ml (GM-CSF, IL-1β, IL-2, IL-4, IL-13, IL-17, IL-18, TNFα) or were present at constant levels and not affected by the influenza virus infection (G-CSF, IFNγ, IL-12, MIP-1β, sCD40L, TGFα) (not shown). Cytokines and chemokines could not be detected in marmosets because of lack of cross-reactivity of reagents for this species.

## Discussion

Cynomolgus and rhesus macaques are both used as animal model in influenza virus research and have been shown to be susceptible to several influenza A virus subtypes, including seasonal and pandemic H1N1, H3N2 and H5N1 [1,4–8,10–14,16–18]. Here, we have performed a head-to-head comparison between these two species using the same stock of a MDCK-propagated swine-origin influenza virus strain A/Mexico/InDRE4487/2009, using the same dose and route of delivery. Although all animals became infected, cynomolgus macaques showed more uniform, higher and prolonged virus production in the trachea and more fever, while clinical symptoms were more outspoken in the rhesus macaque. Furthermore, we observed that common marmosets were also susceptible to infection with the influenza A/Mexico/InDRE4487/2009 pH1N1 virus strain, supporting recent observations obtained with influenza A/California/07/2009 [19].

The viral strain used for this comparison study was chosen on the basis of previously published data showing clear clinical signs, pathology and virus production in the bronchus and lungs in cynomolgus macaques [17,20]. Our observations in cynomolgus macaques are largely in agreement with these studies. Virus was detectable in upper and lower respiratory tract up to day 6 after virus inoculation and similar pathological findings and radiographic scores were obtained. However, clinical symptoms observed in our study were very moderate and largely limited to reduced food intake. Instead, rhesus macaques showed changes in posture, ruffled fur, and strained breathing when active. Nasal discharge and coughing were almost never observed despite the fact that in four of the cynomolgus and in three of the rhesus macaques virus could be detected in the nasal cavity, albeit at relatively low levels in comparison with previously published data [16,18]. Possibly, the intra-bronchial inoculation used here, in contrast to the combined intra-tracheal, oral, intranasal and intraocular administration used by others [16–18,20], may have caused these differences in virus production and clinical symptoms. Surprisingly, virus could also be detected in the blood in several animals by using a sensitive real-time PCR method. We hypothesize that local damage to the lung vasculature may have led to virus entry into the blood, which potentially could result in viral spreading to other organs. We also observed that in some animals virus was present for a prolonged time period, i.e. until day 14, in the upper airways.

It is not clear what causes these species differences. There are some differences in social behavior between the two monkey species, which could lead to different basal stress levels that could have an impact on display of behavior-related symptoms. Importantly, cynomolgus macaques were observed to contain 50- to 74-fold higher levels of sialyl-α-2,6-Gal terminated saccharides in trachea and bronchi as compared to rhesus macaques. This difference in expression is in agreement with the higher virus replication, noted especially in the tracheal swabs and in the lungs. We did not study sialyl-α-2,6-Gal expression in the oro-nasopharynx and nasal mucosa tissues. As indicated above, others have shown high virus replication in the nasal mucosa when the nasal cavity is included as a target during viral challenge. Therefore, it can be assumed that influenza virus receptors are expressed in the nasal cavity. However, there may be more subtle differences between the species. Although interesting, this aspect was not studied by us since the swabs gave variable and generally low virus titers, which makes it difficult to correlate receptor expression with differences in virus production between the species. Unfortunately, we did not have human tissues available to perform the same Western blot analysis and we cannot conclude in which of the two macaque species sialyl-α-2,6-Gal expression most closely resembles the levels present in human upper airway tissue. However, expression of sialyl-α-2,6-Gal receptors on ciliated epithelial cell in the naso-pharynx, trachea and bronchiolo has been demonstrated in humans by studying SNA binding via immunohistochemistry [23–25]. In agreement with our Western blot data on SNA binding ligands in the upper airways of macaques, SNA staining of epithelial cells in nasal turbinates and trachea was recently reported in African green monkeys, although in the bronchus expression was seen primarily in goblet cells [26]. However, we used Western blotting as differences in staining intensity on tissue sections are difficult to quantify.

The use of temperature data loggers that recorded the abdominal temperature every 15 minutes made it possible to precisely record changes in body temperature and to take into account the circadian cycle of each individual animal. Differences in temperature increase between cynomolgus and rhesus macaques were identified that would otherwise have gone unnoticed. Interestingly, besides the rise in temperature that was consistently observed at day 2–3 after infection, an additional fever peak was recorded in some of the animals around day 6–7. A similar second febrile peak was also described in one of the few studies where this was recorded in experimentally-infected humans [27].

In agreement with previous observations on the influenza A/Mexico/InDRE4487/2009 strain [17], we detected a strong increase in IL-6 and MCP-1 in the blood of cynomolgus macaques at day 1 after inoculation, and a more gradual increase in IL-8. Rhesus macaques rather showed an increase in MIP-1α and IL-1Ra, while IL-6 was modestly increased, and MCP-1 remained low. Possibly the relatively lower rise in body temperature seen in rhesus monkeys might be related to this difference in cytokine balance, i.e. lower amounts of the pyrogenic IL-6 and more IL-1Ra to block activity of IL-1. Indeed, IL-6 has been associated with clinical manifestations in humans [28,29], was increased in serum and lungs in pathogenic H5N1 [4] and pathogenic 1918 H1N1 infection in cynomolgus macaques [15] and might therefore be considered as a marker for severity. The rapid induction of IL-6, MCP-1, IL-15 and MIP-1α in serum at day 1 after infection suggests an immediate activation of the macrophage/monocyte/dendritic cell innate immune system [30]. The more gradual increase in IL-8 could indicate that for this cytokine other cells, like fibroblasts or infected epithelial cells are responsible. However, this was not investigated by us. While MCP-1 should especially result in monocyte and dendritic cell activation and recruitment and MIP-1α should rather have an effect on the function of polymorphonuclear cells, the reaction pattern of these cell types in the blood was similar between cynomolgus and rhesus macaques (Fig 7) [30]. IL-15 can lead to NK and CD8 T-cell activation. Indeed we observed that NK cell numbers were transiently reduced in the blood at day 1 after infection, followed by a return of activated HLA-DR expressing NK cells (not shown). Further studies should clarify whether differences do occur in the lungs or bronchial lymph nodes.

As shown by FACS analysis, early activation of CD4 (CD25) and CD8 (CD69) T-cells was more prominent in cynomolgus macaques, while in both species a more gradual induction of CD8 and, to a lesser extent CD4, T-cell proliferation was observed. A similar pattern of proliferation induction was previously reported [31] and was reported to coincide with the formation of influenza-specific IFNγ cytokine responses. Strong decreases in blood lymphocyte as well as granulocyte counts were observed at day 1 and 2 after infection respectively, in both cynomolgus and rhesus macaques, which is in agreement with previous reports in macaques [4] and humans [27].

Recently, the common marmoset, a New World monkey species, was shown to be susceptible to infection with pandemic swine origin H1N1 influenza virus [19]. Using a different pandemic H1N1 strain we confirm these observations. In view of the limited data available in this species, and considering the heterogeneity in virulence and morbidity between genetically similar influenza virus strains isolated during the early stage of the pandemic [17], the evidence presented here supports marmosets as a valid NHP model for influenza A virus infection.

In summary, this study presents the first comparative analysis on susceptibility to influenza A virus infection in three non-human primate species, of which the cynomolgus and rhesus macaque are already used as animal model in influenza research. Using the influenza A/Mexico/InDRE4487/2009 strain we show higher virus replication in the upper airways and fever in cynomolgus relative to rhesus macaques, most probably related to the high abundance of influenza virus receptors in the upper airways of cynomolgus macaques. In addition, we also confirm the susceptibility of common marmosets to influenza virus A infection. These animal models can be useful to decipher the immunological mechanisms that are involved in the early innate control of virus replication. The uniform and high level of virus replication in the throat of cynomolgus macaques, as well as the more pronounced increase in temperature, makes this macaque species more suitable for studying vaccine induced efficacy against infection. Instead rhesus macaques display more clinical symptoms and may be more suitable for disease prevention studies, like therapeutic intervention strategies. However, symptoms associated with upper airway infection, such as nasal discharge, sneezing, excessive salivation, ocular discharge, lacrimation were absent in both species, implying that therapeutic efficacy can only be assessed with regard to symptoms associated with breathing and appetite. Both species have the advantage that larger and more frequent blood samples can be collected for analysis. Similarly, the availability of many cross-reactive reagents allows in-depth analysis of immunological responses. Common marmosets do not have these advantages. However, this species may be preferable for studies with antiviral drugs or therapeutic monoclonal antibodies. Because of the low body weight only small amounts of compound are needed, which may be advantageous in the earlier stages of drug development. In general, influenza infection in the macaque species is comparable to what is seen in humans with regard to the relatively low pathogenicity, fever induction with a second peak at day 6–7 in some animals, and the decrease in lymphocyte and granulocyte count. However, nasal discharge, sneezing and other signs of upper airway infection are not observed.

## Supporting Information

S1 Fig Representative radiographs. (DOCX)

S2 Fig Representative histological images of lung pathology. (DOCX)

S1 Table Virus detection and replication in cynomolgus macaques, rhesus macaques and common marmosets after Mex 4487 infection measured by RT-PCR and culture on MDCK cells. (DOCX)
