# Supplementary material for: Pandemic Swine-Origin H1N1 Influenza Virus Replicates to Higher Levels and Induces More Fever and Acute Inflammatory Cytokines in Cynomolgus versus Rhesus Monkeys and Can Replicate in Common Marmosets
Source: PLoS One. 2015 May 6;10(5):e0126132. doi: 10.1371/journal.pone.0126132 (PMC4422689; doi:10.1371/journal.pone.0126132)
Supplement: S1 Fig — (DOCX) [file pone.0126132.s001.docx]

**
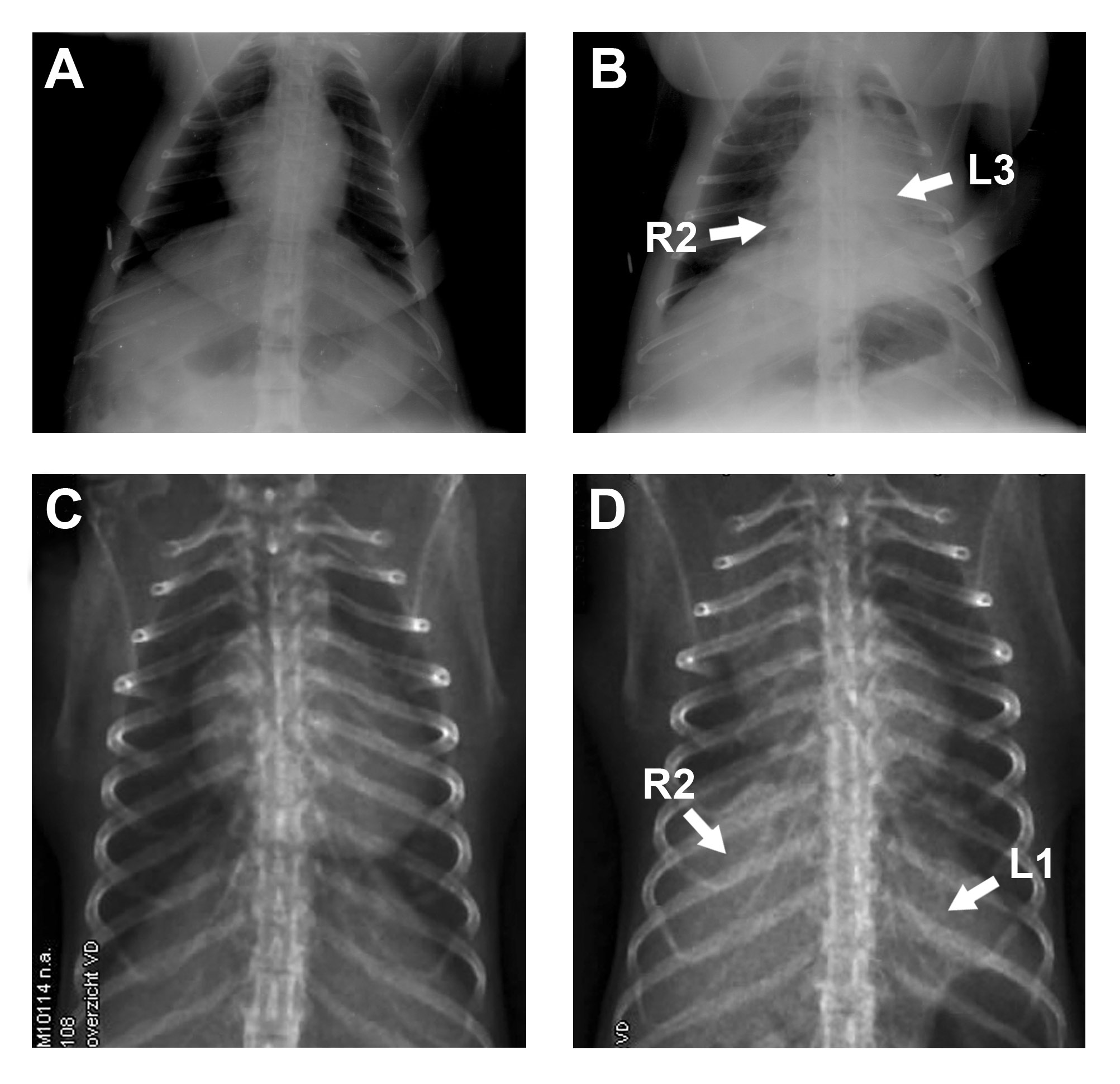
**

**S1 Figure. Representative radiographs**. Showing; A. cynomolgus monkey C4 without any pathology at day 2 post infection and B. animal C4 at day 6 after infection, with grade 2 lung pathology on the right side of the animal (R2, moderate interstitial pulmonary infiltrates, possibly including partial cardiac border effacement and small areas of pulmonary consolidation) and grade 3 lung pathology on the left (L3, pulmonary consolidation as primary lung pathology, often seen as progression from grade 2 lung pathology) and, C. marmoset M6 before infection without any lung pathology and D. animal M6 with grade 2 lung pathology on the right (R2) and grade 1 lung pathology on the left (L1, mild interstitial pulmonary infiltrates) according to Brining et al. [20].
