# Supplementary material for: Pandemic Swine-Origin H1N1 Influenza Virus Replicates to Higher Levels and Induces More Fever and Acute Inflammatory Cytokines in Cynomolgus versus Rhesus Monkeys and Can Replicate in Common Marmosets
Source: PLoS One. 2015 May 6;10(5):e0126132. doi: 10.1371/journal.pone.0126132 (PMC4422689; doi:10.1371/journal.pone.0126132)
Supplement: S2 Fig — (DOCX) [file pone.0126132.s002.docx]

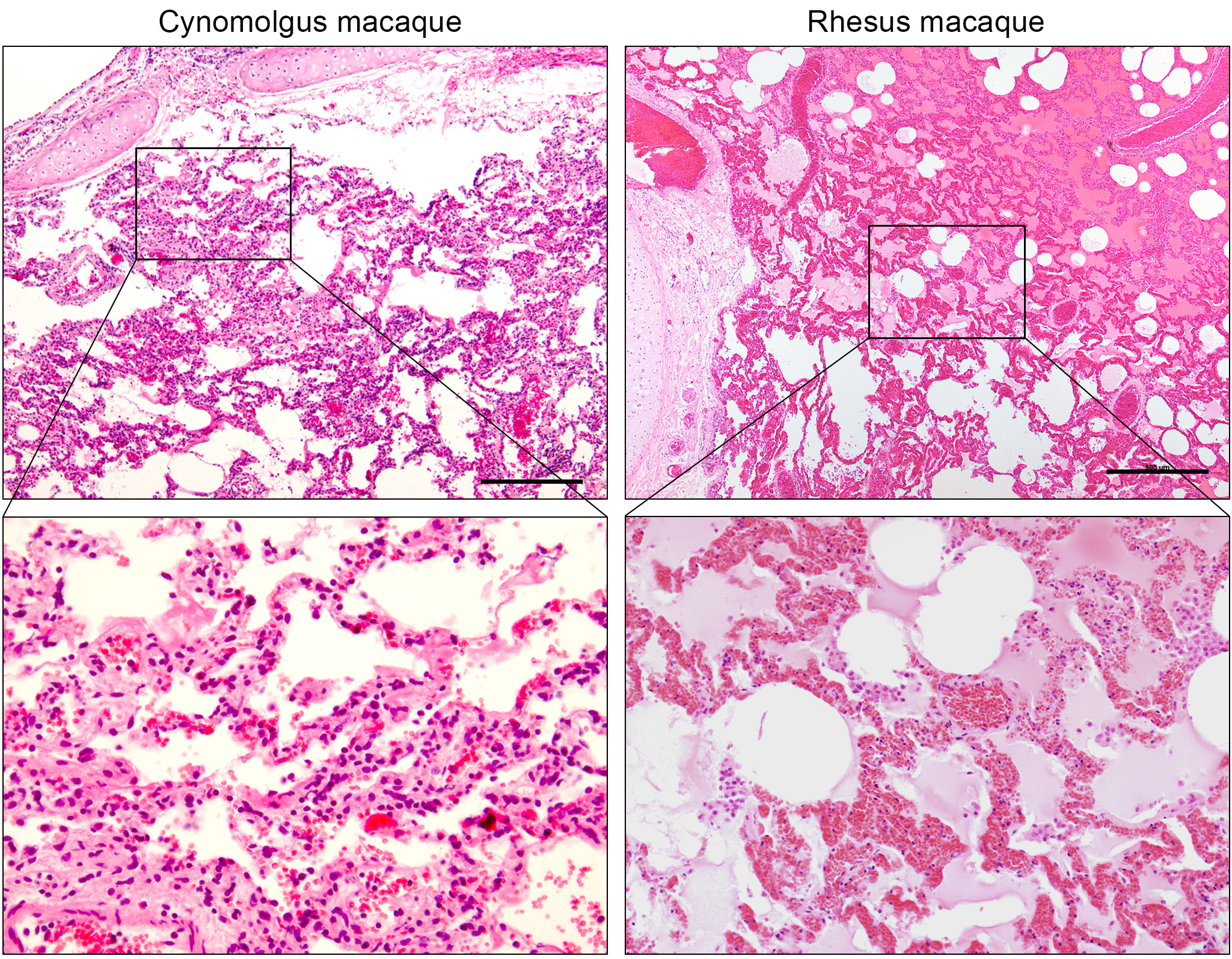


**S2 Figure.** **Representative histological images of lung pathology.** Histogical images (HE staining) of the lung of a cynomolgus macaque (C4, left images) and rhesus macaque (R4, right images) at day 6 post-infection are shown at original magnification x50 (top images) and in detail (lower images). In the cynomolgus macaque an accumulation of inflammatory cells, edema, multifocal epithelial cell loss (necrosis), intra-alveolar hemorrhage is evident, while in the rhesus macaque a marked edema, congestion, intra-alveolar hemorrhage with variable number of inflammatory cells is more prominent. Bar is 500 μM.
