# Supplementary material for: Pandemic Swine-Origin H1N1 Influenza Virus Replicates to Higher Levels and Induces More Fever and Acute Inflammatory Cytokines in Cynomolgus versus Rhesus Monkeys and Can Replicate in Common Marmosets
Source: PLoS One. 2015 May 6;10(5):e0126132. doi: 10.1371/journal.pone.0126132 (PMC4422689; doi:10.1371/journal.pone.0126132)
Supplement: S1 Table — (DOCX) [file pone.0126132.s003.docx]

**S1 Table. Virus detection and replication in cynomolgus macaques, rhesus macaques and common marmosets after Mex4487 influenza virus infection measured by RT- PCR and culture on MDCK cells.**

|  | dpi | 1 | 1 | 2 | 2 | 3 | 3 | 4 | 4 | 6 | 6 | 8 | 8 | 10 | 10 | 14 | 14 |
| --- | --- | --- | --- | --- | --- | --- | --- | --- | --- | --- | --- | --- | --- | --- | --- | --- | --- |
|  |  | PCR | TCID50 | PCR | TCID50 | PCR | TCID50 | PCR | TCID50 | PCR | TCID50 | PCR | TCID50 | PCR | TCID50 | PCR | TCID50 |
| C1 | T | 6,94E+04 | 4,48E+02 | 2,62E+06 | 1,66E+04 | 8,80E+04 | neg |  |  |  |  |  |  |  |  |  |  |
| C2 | T | 3,35E+06 | 1,87E+05 | 2,80E+04 | neg | 1,64E+04 | 1,48E+03 |  |  |  |  |  |  |  |  |  |  |
| C3 | T | 3,18E+06 | 5,64E+04 | 1,20E+05 | 1,36E+02 |  |  | 4,40E+04 | 1,36E+02 | 6,60E+04 | neg |  |  |  |  |  |  |
| C4 | T | 3,07E+04 | neg | 3,38E+05 | 2,96E+02 |  |  | 6,80E+05 | 5,64E+04 | 1,42E+05 | 2,09E+06 |  |  |  |  |  |  |
| C5 | T | 1,03E+05 | 1,36E+02 | 2,60E+04 | neg |  |  | 2,40E+04 | neg | 1,27E+03 | neg | 9,64E+02 | neg | 6,00E+02 | neg | neg | neg |
| C6 | T | 4,22E+05 | 2,52E+04 | 1,60E+05 | 1,36E+02 |  |  | 3,80E+04 | 1,36E+02 | 2,20E+05 | 1,48E+03 | 7,44E+02 | neg | 9,80E+01 | neg | 5,32E+02 | neg |
| C1 | N | neg | 2,00E+02 | neg | neg | 1,26E+05 | 1,00E+03 |  |  |  |  |  |  |  |  |  |  |
| C2 | N | neg | neg | neg | neg | neg |  |  |  |  |  |  |  |  |  |  |  |
| C3 | N | neg | neg | neg | neg |  |  | neg |  | 9,10E+02 | neg |  |  |  |  |  |  |
| C4 | N | neg | neg | neg | neg |  |  | neg |  | neg |  |  |  |  |  |  |  |
| C5 | N | neg | neg | neg |  |  |  | 5,56E+02 | neg | neg |  | neg |  | neg |  | neg |  |
| C6 | N | neg | neg | neg |  |  |  | 9,00E+04 | 4,48E+02 | 1,18E+06 | 1,12E+04 | 1,62E+03 | neg | 3,00E+04 | neg | 6,14E+02 | neg |
| C1 | B | neg |  | neg |  | neg |  |  |  |  |  |  |  |  |  |  |  |
| C2 | B | neg |  | neg |  | 4,06E+02 | 1,12E+03 |  |  |  |  |  |  |  |  |  |  |
| C3 | B | neg |  | neg |  |  |  | 1,80E+03 | 2,24E+02 | neg |  |  |  |  |  |  |  |
| C4 | B | neg |  | neg |  |  |  | neg |  | 2,90E+03 | 2,24E+02 |  |  |  |  |  |  |
| C5 | B | neg |  | neg |  |  |  | 4,50E+02 | 1,12E+03 | 1,90E+04 | 1,12E+03 | neg |  | 1,94E+02 | 1,12E+03 | neg |  |
| C6 | B | neg |  | neg |  |  |  | neg |  | neg |  | neg |  | neg |  | neg |  |
| C1 | L |  |  |  |  | 1,10E+05 | 4,17E+04 |  |  |  |  |  |  |  |  |  |  |
| C2 | L |  |  |  |  | 3,60E+06 | 4,68E+05 |  |  |  |  |  |  |  |  |  |  |
| C3 | L |  |  |  |  |  |  |  |  | 5,70E+03 | 5,01E+02 |  |  |  |  |  |  |
| C4 | L |  |  |  |  |  |  |  |  | 1,80E+05 | 2,09E+05 |  |  |  |  |  |  |
| C5 | L |  |  |  |  |  |  |  |  |  |  |  |  |  |  | 3,91E+02 | neg |
| C6 | L |  |  |  |  |  |  |  |  |  |  |  |  |  |  | neg |  |

Dpi: days post infection, T: tracheal swab, N: nasal swab, B: blood (plasma), L: bronchoalveolar lavage (BAL). C1-6: cynomolgus monkey 1-6. Expressed as: RNA copies or TCID_50_ per swab (for trachea and nose) or RNA copies or TCID_50_ per ml (for blood and BAL). Influenza virus replication was tested on MDCK cells only when a sample was tested virus positive with RT-PCR.

|  | dpi | 1 | 1 | 2 | 2 | 3 | 3 | 4 | 4 | 6 | 6 | 8 | 8 | 10 | 10 | 14 | 14 |
| --- | --- | --- | --- | --- | --- | --- | --- | --- | --- | --- | --- | --- | --- | --- | --- | --- | --- |
|  |  | PCR | TCID50 | PCR | TCID50 | PCR | TCID50 | PCR | TCID50 | PCR | TCID50 | PCR | TCID50 | PCR | TCID50 | PCR | TCID50 |
| R1 | T | neg |  | neg |  | 4,42E+02 | neg |  |  |  |  |  |  |  |  |  |  |
| R2 | T | 1,11E+04 | neg | 3,72E+04 | neg | 1,08E+06 | 8,34E+04 |  |  |  |  |  |  |  |  |  |  |
| R3 | T | neg |  | 6,45E+03 | neg |  |  | neg |  | neg |  |  |  |  |  |  |  |
| R4 | T | 3,08E+03 | 1,36E+02 | 1,46E+03 | neg |  |  | 5,76E+02 | neg | neg |  |  |  |  |  |  |  |
| R5 | T | neg |  | neg |  |  |  | neg |  | neg |  | neg |  | neg |  | neg |  |
| R6 | T | 5,27E+04 | neg | 6,49E+03 | neg |  |  | neg |  | neg |  | neg |  | neg |  | neg |  |
| R1 | N | neg |  | 4,74E+02 | neg | neg |  |  |  |  |  |  |  |  |  |  |  |
| R2 | N | neg |  | neg |  | neg |  |  |  |  |  |  |  |  |  |  |  |
| R3 | N | neg |  | neg |  |  |  | neg |  | neg |  |  |  |  |  |  |  |
| R4 | N | neg |  | 6,48E+02 | neg |  |  | neg |  | neg |  |  |  |  |  |  |  |
| R5 | N | 6,52E+02 | neg | neg |  |  |  | neg |  | neg |  | neg |  | neg |  | neg |  |
| R6 | N | neg |  | neg |  |  |  | neg |  | neg |  | neg |  | neg |  | neg |  |
| R1 | B | neg |  | neg |  | 3,34E+02 | neg |  |  |  |  |  |  |  |  |  |  |
| R2 | B | neg |  | neg |  | neg |  |  |  |  |  |  |  |  |  |  |  |
| R3 | B | neg |  | neg |  |  |  | neg |  | neg |  |  |  |  |  |  |  |
| R4 | B | neg |  | neg |  |  |  | neg |  | neg |  |  |  |  |  |  |  |
| R5 | B | neg |  | neg |  |  |  | neg |  | neg |  | neg |  | neg |  | neg |  |
| R6 | B | neg |  | neg |  |  |  | neg |  | neg |  | neg |  | neg |  | neg |  |
| R1 | L |  |  |  |  | 8,64E+05 | 2,51E+03 |  |  |  |  |  |  |  |  |  |  |
| R2 | L |  |  |  |  | 1,16E+03 | 5,62E+03 |  |  |  |  |  |  |  |  |  |  |
| R3 | L |  |  |  |  |  |  |  |  | 3,58E+02 | neg |  |  |  |  |  |  |
| R4 | L |  |  |  |  |  |  |  |  | neg |  |  |  |  |  |  |  |
| R5 | L |  |  |  |  |  |  |  |  |  |  |  |  |  |  | neg |  |
| R6 | L |  |  |  |  |  |  |  |  |  |  |  |  |  |  | neg |  |

Dpi: days post infection, T: tracheal swab, N: nasal swab, B: blood (plasma), L: bronchoalveolar lavage (BAL). R1-6: rhesus monkey 1-6.

Expressed as: RNA copies or TCID_50_ per swab (for trachea and nose) or RNA copies or TCID_50_ per ml (for blood and BAL). Influenza virus replication was tested on MDCK cells only when a sample was tested virus positive with RT-PCR.

|  | dpi | 1 | 1 | 2 | 2 | 3 | 3 | 4 | 4 | 6 | 6 | 8 | 8 | 10 | 10 | 14 | 14 |
| --- | --- | --- | --- | --- | --- | --- | --- | --- | --- | --- | --- | --- | --- | --- | --- | --- | --- |
|  |  | PCR | TCID50 | PCR | TCID50 | PCR | TCID50 | PCR | TCID50 | PCR | TCID50 | PCR | TCID50 | PCR | TCID50 | PCR | TCID50 |
| M1 | T | neg |  | neg |  | neg |  |  |  |  |  |  |  |  |  |  |  |
| M2 | T | neg |  | neg |  | neg |  |  |  |  |  |  |  |  |  |  |  |
| M3 | T | neg |  | neg |  |  |  | neg |  | 1,23E+04 | 1,36E+02 |  |  |  |  |  |  |
| M4 | T | neg |  | neg |  |  |  | neg |  | 3,35E+04 | 2,24E+03 |  |  |  |  |  |  |
| M5 | T | neg |  | neg |  |  |  | neg |  | neg |  | neg |  | neg |  | neg |  |
| M6 | T | neg |  | neg |  |  |  | neg |  | neg |  | 8,94E+07 | 6,18E+05 | neg |  | neg |  |
| M1 | B | neg |  | neg |  | neg |  |  |  |  |  |  |  |  |  |  |  |
| M2 | B | neg |  | neg |  | neg |  |  |  |  |  |  |  |  |  |  |  |
| M3 | B | neg |  | neg |  |  |  | neg |  | neg |  |  |  |  |  |  |  |
| M4 | B | neg |  | neg |  |  |  | neg |  | neg |  |  |  |  |  |  |  |
| M5 | B | neg |  | neg |  |  |  | neg |  | neg |  | neg |  | neg |  | neg |  |
| M6 | B | neg |  | neg |  |  |  | neg |  | neg |  | neg |  | neg |  | neg |  |

Dpi: days post infection, T: tracheal swab, B: blood (plasma). M1-6: marmoset 1-6. Expressed as: RNA copies or TCID_50_ per swab (trachea) or RNA copies or TCID_50_ per ml (blood). Influenza virus replication was tested on MDCK cells only when a sample was tested virus positive with RT-PCR.
